# Anxiety and depression-like behaviours are more frequent in aged male mice conceived by ART compared with natural conception

**DOI:** 10.1530/REP-21-0175

**Published:** 2021-10-04

**Authors:** Ning-Xin Qin, Yi-Ran Zhao, Wei-Hui Shi, Zhi-Yang Zhou, Ke-Xin Zou, Chuan-Jin Yu, Xia Liu, Ze-Han Dong, Yi-Ting Mao, Cheng-Liang Zhou, Jia-Le Yu, Xin-Mei Liu, Jian-Zhong Sheng, Guo-Lian Ding, Wen-Long Zhao, Yan-Ting Wu, He-Feng Huang

**Affiliations:** 1 The International Peace Maternity and Child Health Hospital, School of Medicine, Shanghai Jiao Tong University, Shanghai, China; 2 Shanghai Key Laboratory of Embryo Original Diseases, Shanghai, China; 3 Hospital of Obstetrics and Gynecology, Fudan University, Shanghai, China; 4 Department of Assisted Reproductive Medicine, Shanghai First Maternity and Infant Hospital, School of Medicine, Tongji University, Shanghai, China; 5 Department of Pathology and Pathophysiology, Zhejiang University School of Medicine, Hangzhou, Zhejiang, China; 6 The Key Laboratory of Reproductive Genetics (Zhejiang University), Ministry of Education, Zhejiang University School of Medicine, Hangzhou, Zhejiang, China

## Abstract

The number of children born after assisted reproductive technology (ART) is accumulating rapidly, and the health problems of the children are extensively concerned. This study aims to evaluate whether ART procedures alter behaviours in male offspring. Mouse models were utilized to establish three groups of offspring conceived by natural conception (NC), *in vitro* fertilization and embryo transfer (IVF-ET), and frozen-thawed embryo transfer (IVF-FET), respectively. A battery of behaviour experiments for evaluating anxiety and depression levels, including the open field test (OFT), elevated plus maze (EPM) test, light/dark transition test (L/DTT), tail suspension test (TST), forced swimming test (FST), and sucrose preference test (SPT) was carried out. Aged (18 months old), but not young (3 months old), male offspring in the IVF-ET and IVF-FET groups, compared with those in the NC group, exhibited increased anxiety and depression-like behaviours. The protein expression levels of three neurotrophins in PFC or hippocampus in aged male offspring from the IVF-ET and IVF-FET groups reduced at different extent, in comparison to NC group. RNA sequencing (RNA-Seq) was performed in the hippocampus of 18 months old offspring to further explore the gene expression profile changes in the three groups. KEGG analyses revealed the coexisted pathways, such as PI3K-Akt signalling pathway, which potentially reflected the similarity and divergence in anxiety and depression between the offspring conceived by IVF-ET and IVF-FET. Our research suggested the adverse effects of advanced age on the psychological health of children born after ART should be highlighted in the future.

## Introduction

Assisted reproductive technology (ART), mainly including *in vitro* fertilization and embryo transfer (IVF-ET), frozen-thawed embryo transfer (FET), has been widely used for infertility treatment since the first baby was born from IVF-ET in 1978 (Steptoe & Edwards 1978). The health problems of children born after ART are concerned extensively (Hart & Norman 2013*a*, *b*, Turkgeldi *et al.* 2016, Berntsen *et al.* 2019). Previous studies have suggested adverse effects of ART on the offspring such as heightened risks of autism and intellectual disability, arterial hypertension, cancer, and metabolic dysfunctions during childhood and adolescence (Sandin *et al.* 2013, Meister *et al.* 2018, Hargreave *et al.* 2019, Cui *et al.* 2020). Furthermore, studies using ART mouse models have shown the potential risks of many diseases, especially glucose metabolism dysfunction and cardiovascular diseases, in the adult and aged offspring (Watkins *et al.* 2007, Scott *et al.* 2010, Chen *et al.* 2014, Donjacour *et al.* 2014, Schenewerk *et al.* 2014, Rexhaj *et al.* 2015, Cerny *et al.* 2017, Wang *et al.* 2018, Zheng *et al.* 2018, Aljahdali *et al.* 2020). However, there are still limited studies focused on psychiatry on ART-conceived offspring.

In modern society, mental disorders are common problems concerned extensively (Barbeito *et al.* 2021). Anxiety and depression are among the most frequent mental disorders with an approximated prevalence of 7.3 and 4.7%, respectively, ultimately leading to destroy social ability and work enthusiasm of patients, increase suicide rate in the population, and cause serious health and economic burden in the global population (Baxter *et al.* 2013, Ferrari *et al.* 2013). Timely diagnosis and prevention of anxiety and depression enable to reduce the medical cost and burden, slow down the development of the diseases, and improve the prognosis (Garber *et al.* 2016, Werner-Seidler *et al.* 2017, Faye *et al.* 2018, Salazar de Pablo *et al.* 2021). However, studies regarding anxiety and depression status for ART-conceived offspring are still lacking. Meantime, it is difficult to assess anxiety and depression in human beings conceived by ART in adulthood and old age, accounting for a short time span from the first application of the technologies. Due to the life span of mice is shorter than that of humans, the mouse model is an effective approach to assess the long-term effects on offspring conceived by ART (Vrooman & Bartolomei 2017, Duranthon & Chavatte-Palmer 2018, Sharpe 2018).

There are many classical methods to evaluate anxiety and depression levels in mouse (Pollak *et al.* 2010, Harro 2019, Himanshu *et al.* 2020, Moulin *et al.* 2021, Pentkowski *et al.* 2021). For example, the open field test (OFT), elevated plus maze (EPM), and light/dark transition test (L/DTT) were carried out to assess the anxiety behaviours in the ART-conceived mice. The tail suspension test (TST), forced swimming test (FST), and sucrose preference test (SPT) were applied to assess depression behaviours. Basic locomotion was recorded in the OFT to assess mouse motility. As neuropsychiatric symptoms are always linked to cognition impairment, spatial learning and memory ability were evaluated using the Morris water maze (MWM) (Unal & Canbeyli 2019). It is well known that sex bias between males and females was common in neuroethology. There were previous studies reported that fluctuating levels of gonadal steroids and inconsistent oestrous cycle among each female mouse would interfere with the accuracy of behavioural results (Carrier *et al.* 2015, Boivin *et al.* 2017, Oyola & Handa 2017, Andreae & Basson 2018, Datta *et al.* 2019, Gerhard *et al.* 2021, Mossa & Manzini 2021). Secondly, loss of reproductive competence and alteration of hormone levels in old female mice are more apparent in old male mice at 18 months old. These physiological phenomena also exist in humans. These factors mentioned above suggest more confounders would be brought in our result if we evaluate the female offspring. Thus, we only performed behavioural tests in male offspring.

The hippocampus and prefrontal cortex (PFC) are two crucial brain regions strongly involved in anxiety and depression-like behaviours in rodents (Snyder *et al.* 2011, Kheirbek *et al.* 2012, Hiser & Koenigs 2018, Hare & Duman 2020). Decreased volume, neurogenesis, and altered neuronal apoptosis in the hippocampus have been implicated in the pathogenesis of anxiety and depression (Snyder *et al.* 2011, Kheirbek *et al.* 2012, Mineur *et al.* 2013). The PFC is a key node of cortical and subcortical networks that subserve psychological functions linked to psychopathology (Hiser & Koenigs 2018). Brain-derived neurotrophic factor (BDNF), glial cell-derived neurotrophic factor (GDNF), and nerve growth factor (NGF) are three important neurotrophins involved in neurogenesis and pathophysiology of many nervous system diseases (Angelucci *et al.* 2004, Allen *et al.* 2013, de Azevedo Cardoso *et al.* 2014, Castren & Monteggia 2021). The reduction of neurotrophins in the hippocampus and PFC was one of the major pathological features in anxiety and depression (Thoenen 1995, Autry & Monteggia 2012, Kristiansen & Ham 2014, Notaras *et al.* 2015, Ayanlaja *et al.* 2018, Koo *et al.* 2019). These three neurotrophins play essential roles in nervous system development and function by promoting nerve growth, neurological development, and neuronal plasticity (Thoenen 1995, Kalueff *et al.* 2006, Martinowich *et al.* 2007).

The aim of the study was to assess the anxiety and depression levels in ART-conceived male mice offspring in adulthood and old age. Importantly, ART-conceived male mice exhibited increased anxiety and depression behaviours in old age compared with offspring conceived by natural conception, rather than in adulthood. Decreased neurotrophin protein expression levels in the hippocampus and PFC of mice in the IVF-ET and IVF-FET groups, compared with NC group, were in concordance with the behavioural results.

## Materials and methods

### Mouse model and experimental design

**Figure 1:**
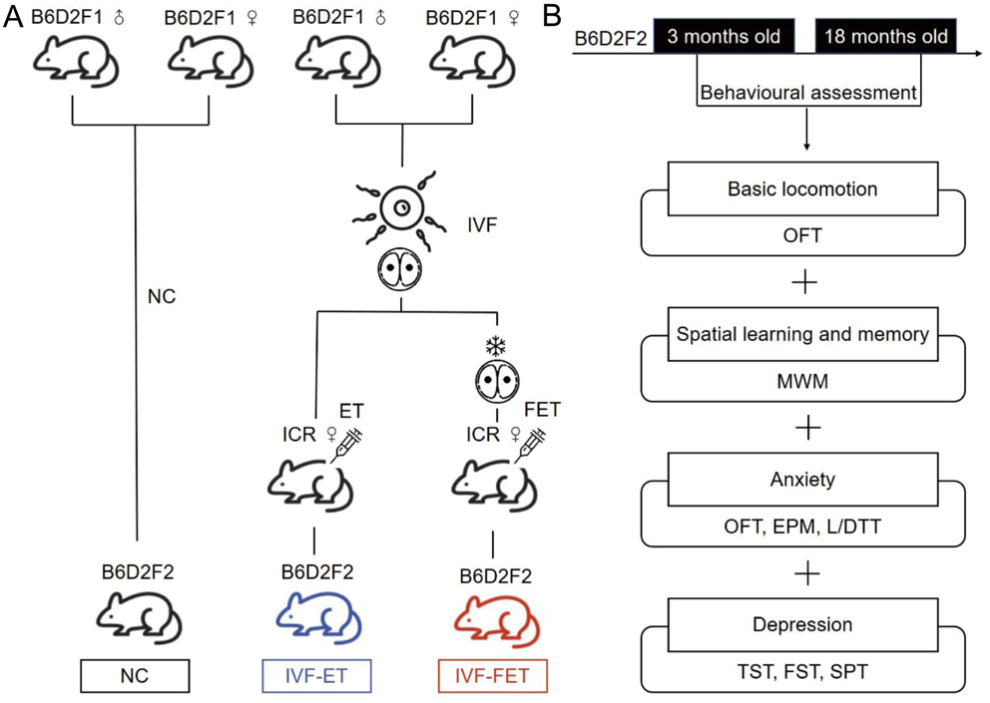
Experimental design. (A) The male mice offspring were divided into NC, IVF-ET, and IVF-FET groups. (B) Behavioural experiments were performed in the male mice offspring at 3 and 18 months old.

All experimental procedures with mice were approved by the Shanghai Model Organisms Center ethical committee in animal research (IACUC approval number: 2019-0002). Virgin 6- to 8-week-old B6D2F1/J (C57B6L/J x DBA2/J) female mice, adult B6D2F1/J males, 8-week-old ICR females, and adult vasectomized ICR males were used. All animals were housed in the same room maintained under a constant 12 h light: 12 h darkness cycle at 21–23 °C, with free access to food and water. The overall processes in this study were followed by the flowchart in Fig. 1. Mice offspring were divided into three groups according to the different approaches they conceived by (Fig. 1A). The natural conception (NC) group is comprised of male mice offspring conceived by natural mating and delivery. Pseudo-pregnant females ICR mice were obtained by mating with vasectomized male mice. Fresh two-cell embryos or embryos after cryopreservation in the liquid nitrogen for 1 week and thawed for 2 h were transferred to pseudo-pregnant mice. The offspring born after IVF-ET or IVF-FET were referred as to IVF-ET group and IVF-FET group, respectively. Two-cell embryos in IVF-ET and IVF-FET groups were obtained by standard B6D2F1 × B6D2F1 IVF. The IVF, embryo transfer, embryo frozen, and thawed procedures have been described previously (Qin *et al.* 2021). The offspring were weaned and separated by sex until postnatal day 20.

### Behavioural assessments

All behavioural assays were performed when the offspring were 3 and 18 months old (*n*  = 7 male offspring/group/age, Fig. 1B). Behavioural tests were recorded by Ethovision XT 13 software (Noldus Information Technology, Netherlands) from 9:00 to 16:00 h, and data were analysed in a blinded manner. Mice were handled for 3 days and habituated to the testing room for 30 min before performing the behavioural experiments. The apparatuses were cleaned with 70% ethanol and water between trials in all experiments.

#### Open field test (OFT)

The OFT experiment was widely used to assess basic locomotion and anxiety behaviours in rodents (Prut & Belzung 2003). The OFT was conducted using a chamber (40 cm × 40 cm) to determine the general locomotor activity of mice. After placing each mouse in the same corner into the chamber, the behaviour of the mouse was recorded by a camera for 15 min. The centre zone was defined as 20 cm × 20 cm. The total distance, the entry times in the centre zone, the distance, and time spent in the centre zone were indicated the locomotor activity and anxiety level of mice.

#### Morris water maze (MWM)

Spatial learning and memory abilities were assessed by MWM (Puzzo *et al.* 2014). It is mainly composed of a circular pool with a diameter of 1.6 m and a height of 0.6 m, filled with 0.4 m of 18–26°C water. The training and test procedures were carried out as previously described (Brandeis *et al.* 1989). The quadrant with platform was termed as the 'target quadrant'. The latency to platform hidden under water and the distance in the target quadrant were recorded.

#### Elevated plus maze (EPM) test

The EPM was performed to assess anxiety behaviours in the offspring (Walf & Frye 2007). Mice were placed in the centre area of an EPM (arms are 35 × 7 cm, with 150 mm-high black walls on the closed arms), and the trajectories of mice were recorded with a video camera for 5 min. The total distance travelled and the distance, entry times, and time spent in the open arms were automatically quantified by the tracking software.

#### Light/dark transition test (L/DTT)

The L/DTT was performed to assess anxiety level in the offspring, as previously reported (Crawley & Goodwin 1980). Briefly, the apparatus for L/DTT was a two-compartment box (210 × 420 × 250 mm) through a small door. One chamber was brightly illuminated (300 lx, bright chamber), while the other chamber had black plastic walls and was dark (2 lx). The mouse was placed in the dark compartment and given free access to the light compartment for 10 min. The distance travelled and time spent in the light chamber and entries into light and dark chambers were recorded.

#### Tail suspension test (TST) and forced swimming test (FST)

The TST and FST were carried out to assess depression behaviours in the offspring. The TST and FST were performed according to the previous report (Steru *et al.* 1985, Yankelevitch-Yahav *et al.* 2015). In TST, the mice were suspended on an apparatus by their tails individually and recorded for 6 min. In FST, mice were placed in a transparent cylinder filled with 18 cm height of 18–26°C water and videotaped for 6 min. The animal behaviours in the last 4 min were analysed, and the time of struggle (escape-related behaviour) in blind was quantified for both TST and FST. Immobility time = 240 s – struggle time.

#### Sucrose preference test (SPT)

The SPT was performed to assess depression behaviours in the offspring (Liu *et al.* 2018). During the SPT, the mice were individually housed. On the first day of the test, the mice were given two bottles contained 1% sucrose and pure water, respectively. After that, the mice were deprived of drink for 12 h. And then, two bottles that contained 1% sucrose or water were resupplied and switched the location for each other. Twenty-four hours later, the weights of the bottles were measured. The sucrose preference index was calculated using the equation (Δweight of sucrose)/(Δweight of sucrose + Δweight of water) × 100.

### Tissue collection and Western blotting

All mice in each group were sacrificed by cervical dislocation after anaesthetized at the age of 3 months and 18 months by i.p. injection 1.25% tribromoethanol (M2910, Nanjing Aibei Biotechnology Co., Ltd, Nanjing, China) after completion of behavioural assessment. Hippocampal and PFC tissues were isolated immediately from the brains of offspring and frozen directly in liquid nitrogen. Half of the hippocampus and PFC from each mouse was used for RNA extraction, and the other half for protein extraction. Protein was extracted using RIPA lysis buffer (P0013B; Beyotime, Wuhan, China). Samples were separated using SDS-PAGE at 80–120 V for 90 min and transferred onto PVDF membranes at 220 mA for 30 min. The primary antibodies included BDNF antibody (1:2000, ab108319, Abcam ), GDNF antibody (1:1000, ab176564, Abcam), NGF antibody (1:1000, ab52918, Abcam), and GAPDH antibody (1:5000, 10494-1-AP, ProteinTech Group, Inc., Chicago, IL, USA). Primary antibodies were incubated overnight at 4°C, and secondary antibodies were incubated for 1 h at room temperature. The signals were visualized by an ECL system (Amersham Imager 600, GE Healthcare Life Sciences ).

### RNA sequencing and quantitative real-time PCR

Total RNA was isolated from the hippocampus using TRIzol reagent (15596026, Invitrogen). The quality of total RNA was confirmed by agarose gel electrophoresis, and RNA was used for the construction of cDNA library. RNA sequencing (RNA-Seq) was performed in the hippocampus of 18 months old offspring. The sequencing was conducted on an Illumina Nova6000 system and performed by Genergy biological technology Co., Ltd. (Shanghai, China). The reads were trimmed and then mapped to the entire genome. Differential expression genes (DEGs) were determined by fold change > 1 and *P* value < 0.05. GO and KEGG pathway enrichment analyses were performed. The Venn diagram was drawn online (http://jvenn.toulouse.inra.fr/app/example.html) (Bardou *et al.* 2014).

Total RNA was reverse transcribed using a PrimeScript RT reagent Kit with gDNA Eraser (RR047A, Takara, Japan). The TB green Premix EX Taq kit (RR420A, Takara, Japan) was used for PCR. Quantitative real-time PCR (qRT-PCR) reactions were run on Applied Biosystems QuantStudio 7 Flex PCR systems (Thermo Fisher Scientific Inc.). The primer sequences were listed in Supplementary Table 1 (see section on supplementary materials given at the end of this article).

### Statistical analysis

Statistical analyses were conducted using GraphPad Prism 8.0. Data are presented as the mean ± s.e.m. Statistical analyses were performed by non-parametric Kruskal–Wallies test followed by Mann–Whitney test for behavioural results or one-way ANOVA with Tukey’s test for *post hoc* comparisons for qPCR and Western blotting results. Significance was set at *P* < 0.05.

## Results

### Young male offspring conceived by ART did not show any alteration in anxiety and depression tests

At the age of 3 months, the mice in the IVF-ET and IVF-FET groups displayed similar locomotion with NC group in the OFT (Supplementary Fig. 1). Meanwhile, no differences in performance were observed in the MWM among three groups (Supplementary Fig. 1), indicating similar spatial learning abilities. In the EPM test, mice in the three groups spent similar time and distance in the open arms (Supplementary Fig. 1). There were no differences in the immobile time in TST among the three groups (Supplementary Fig. 1). Taken together, the results showed that male mice conceived by ART did not show any alteration in anxiety and depression tests at the age of 3 months.

### Aged male offspring conceived by ART displayed increased anxiety-like behaviours

**Figure 2:**
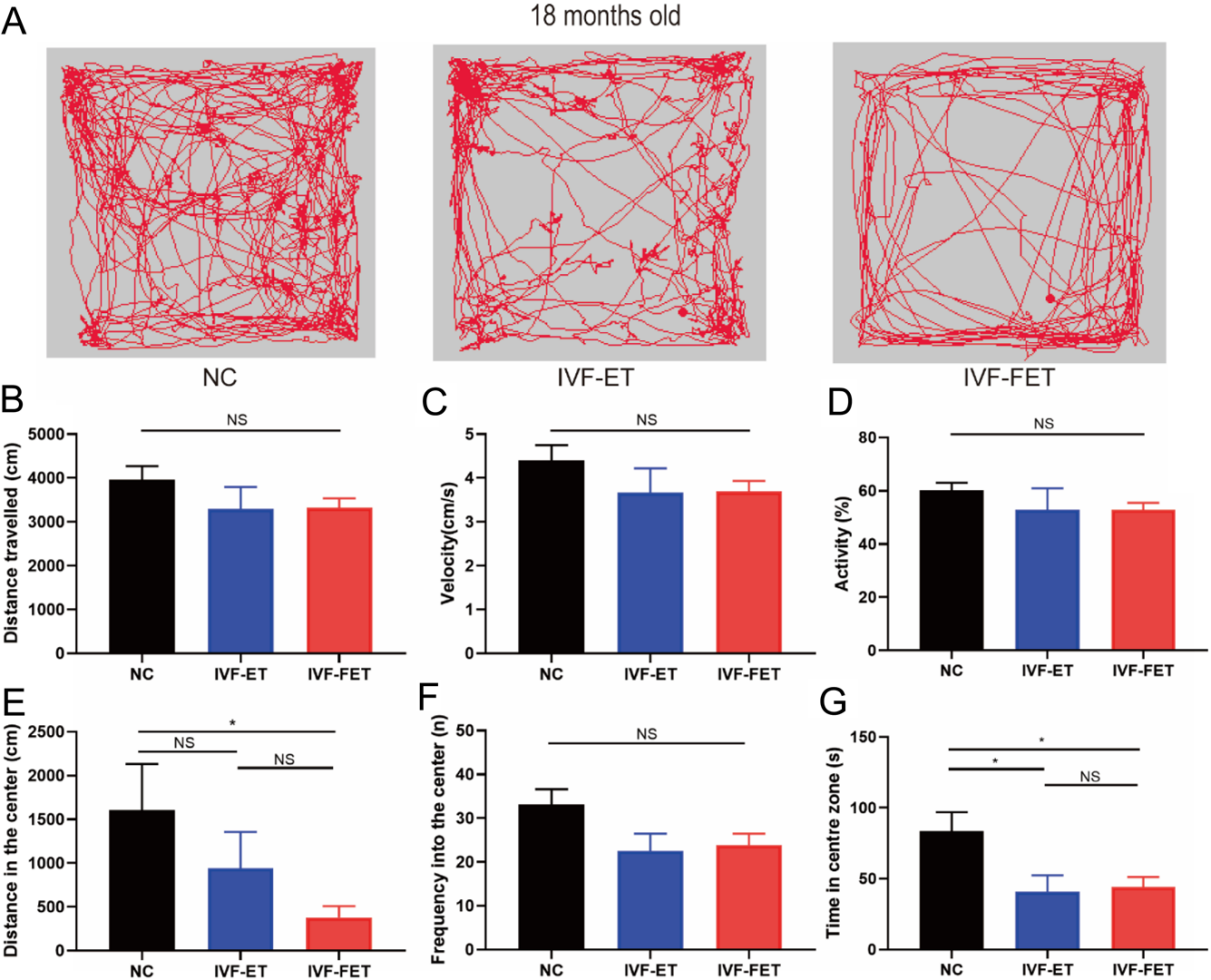
Open field test (OFT) results of male mice offspring at 18 months old. (A) Representative trajectory diagrams in the OFT for the three groups (NC, IVF-ET, and IVF-FET). (B) Total distance travelled in the OFT. (C) Average velocity in the OFT. (D) Percentage of activity in the OFT. (E, F, and G) The distance (E), time (F), and entries (G) in the centre zone. All data are presented as the mean ± s.e.m. (*n*  = 7 mice per group). NS: *P >* 0.05, *P < 0.05.

Figure 2A displays the representative trajectory diagrams in the OFT for the male mice offspring at 18 months old. At the age of 18 months, mice in the three groups travelled similar distances and moved with similar velocities in the OFT (Fig. 2B and C). The percentage of activity also showed no significant differences (Fig. 2D). Similarly, there were no significant differences in the MWM on either training days or test days at the age of 18 months (Supplementary Fig. 2). Compared with NC-conceived mice, ART-conceived mice displayed similar locomotion and spatial learning abilities.

There was a significant difference in distance travelled in the centre zone in the IVF-FET group compared with the NC group (Fig. 2E). Although no differences were observed in the frequency of entering into the centre zone among the three groups (Fig. 2F), there was a significant difference in distance travelled in the centre zone between the NC and IVF-FET groups (Fig. 2G). Less time and distance spent in the centre zone indicated increased anxiety-like behaviours in aged male offspring conceived by IVF-FET.

**Figure 3:**
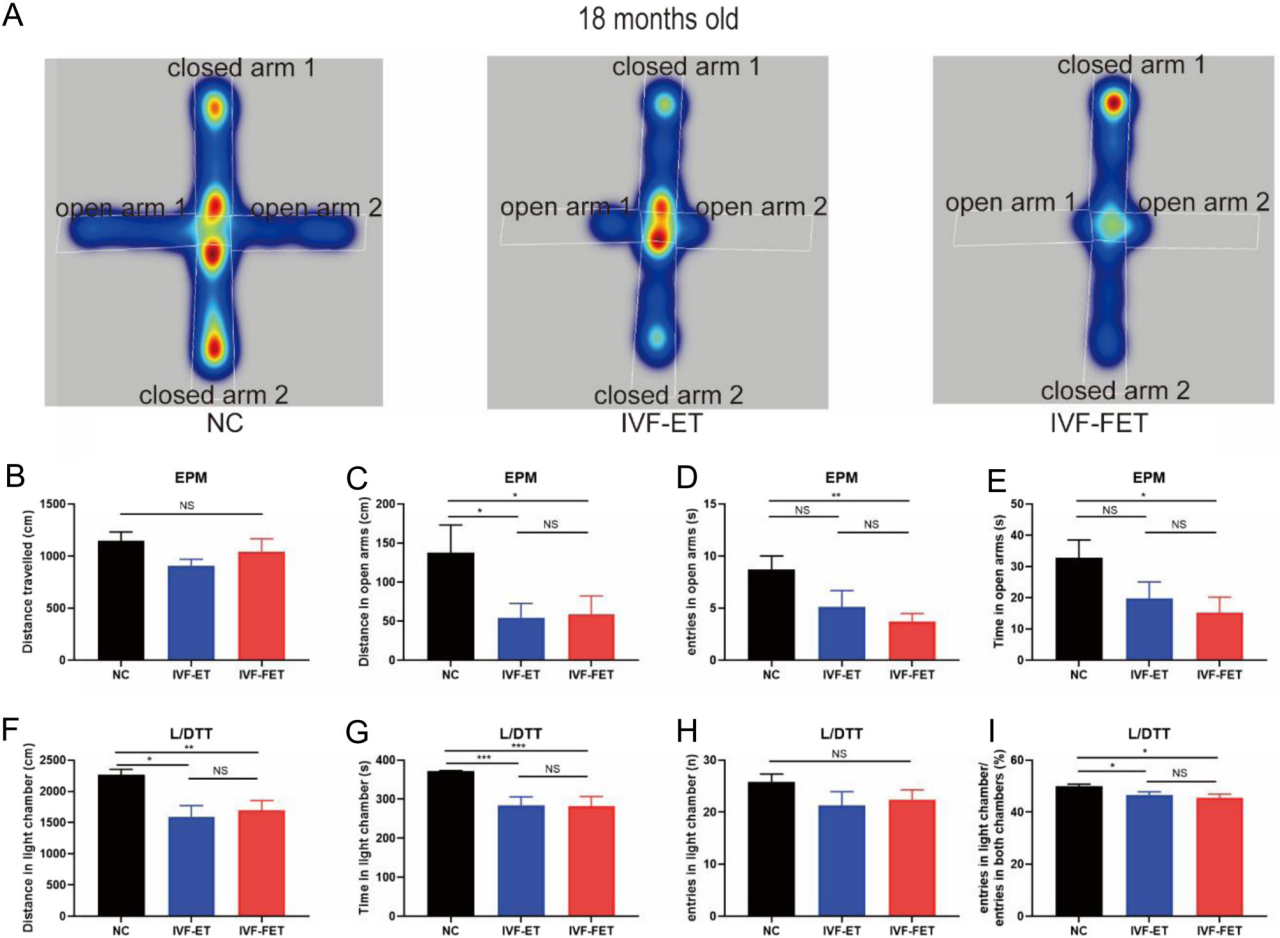
Elevated plus maze (EPM) and light/dark transition test (L/DTT) results of male mice offspring at 18 months old. (A) Representative heatmaps in the EPM for the three groups (NC, IVF-ET, and IVF-FET). (B) Total distance travelled in open and closed arms. (C, D, and E) Distance (C), entries (D), and time (E) in open arms. (F, G, and H) Distance (F), time (G), and entries (H) in the light chamber in the L/DTT. (I) The proportion of entries into the light chamber relative to all entries into both sides. All data are presented as the mean ± s.e.m. (*n* = 7 mice per group). NS: *P* > 0.05, *P < 0.05, **P < 0.01, ***P < 0.001.

Moreover, EPM and L/DTT were performed to further assess the anxiety levels for mice at 18 months old. Representative heatmaps for activity in the EPM are shown in Fig. 3A. In the EPM results, no significant differences were detected in the total distance (Fig. 3B). The mice in the IVF-ET group travelled less distance than these in the NC group, and similar distance compared with the IVF-FET group (Fig. 3C). The results also revealed significant differences in distance travelled, entries into, and time spent in the open arms between the IVF-FET group and the NC group (Fig. 3C, D, and E). The mice in the IVF-ET and IVF-FET groups exhibited increased anxiety-like behaviours with varying degrees of severity for less distance, entries, and time in the open arms.

In the L/DTT, aged mice in the IVF-ET and IVF-FET groups travelled less distance and spent less time in the light chamber than those in the NC group (Fig. 3F and  G). Meanwhile, there was no significant difference in distance and time in the light chamber between the IVF-ET and IVF-FET groups. Although mice in the three groups entered into the light chamber at similar times, the ratio of entries into the light chamber in the IVF-ET and IVF-FET groups seemed to be lower than those in the NC group (Fig. 3H and  I). These data furtherly suggested that aged male offspring conceived by ART displayed anxiety-like behaviours.

### Increased depression-like behaviours in aged male offspring conceived by ART

**Figure 4:**
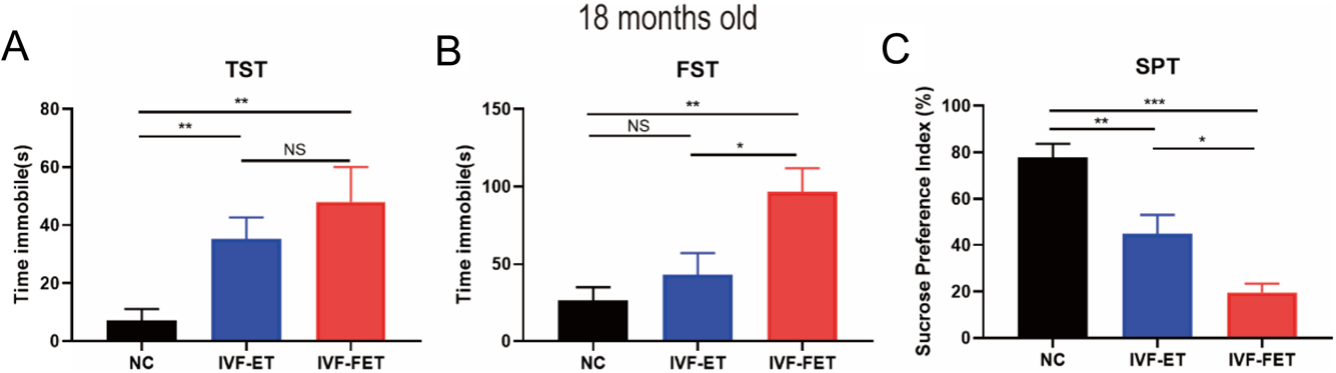
Tail suspension test (TST), force swimming test (FST), and sucrose preference test (SPT) results of male mice offspring at 18 months old. (A and B) Immobility time in the TST (A) and FST (B). (C) Sucrose preference index in SPT. All data are presented as the mean ± s.e.m. (*n* = 7 mice per group) NS: *P* > 0.05, *P < 0.05, **P < 0.01, ***P < 0.001.

For 18 months old mice, there was a significant increase in immobility time in both the TST and FST in the IVF-ET and IVF-FET groups compared with those in the NC group (Fig. 4A and  B), indicating increased depression-like behaviours in the aged male offspring conceived by ART. The sucrose preference index from the SPT also showed a similar trend-wise in the TST and FST (Fig. 4C). Furthermore, the male offspring in the IVF-FET group exhibited more severe depression level than those in the IVF-ET group in the FST and SPT.

### Decreased neurotrophin protein expression levels in the hippocampus and PFC of aged male mice offspring in the IVF-ET and IVF-FET groups

**Figure 5:**
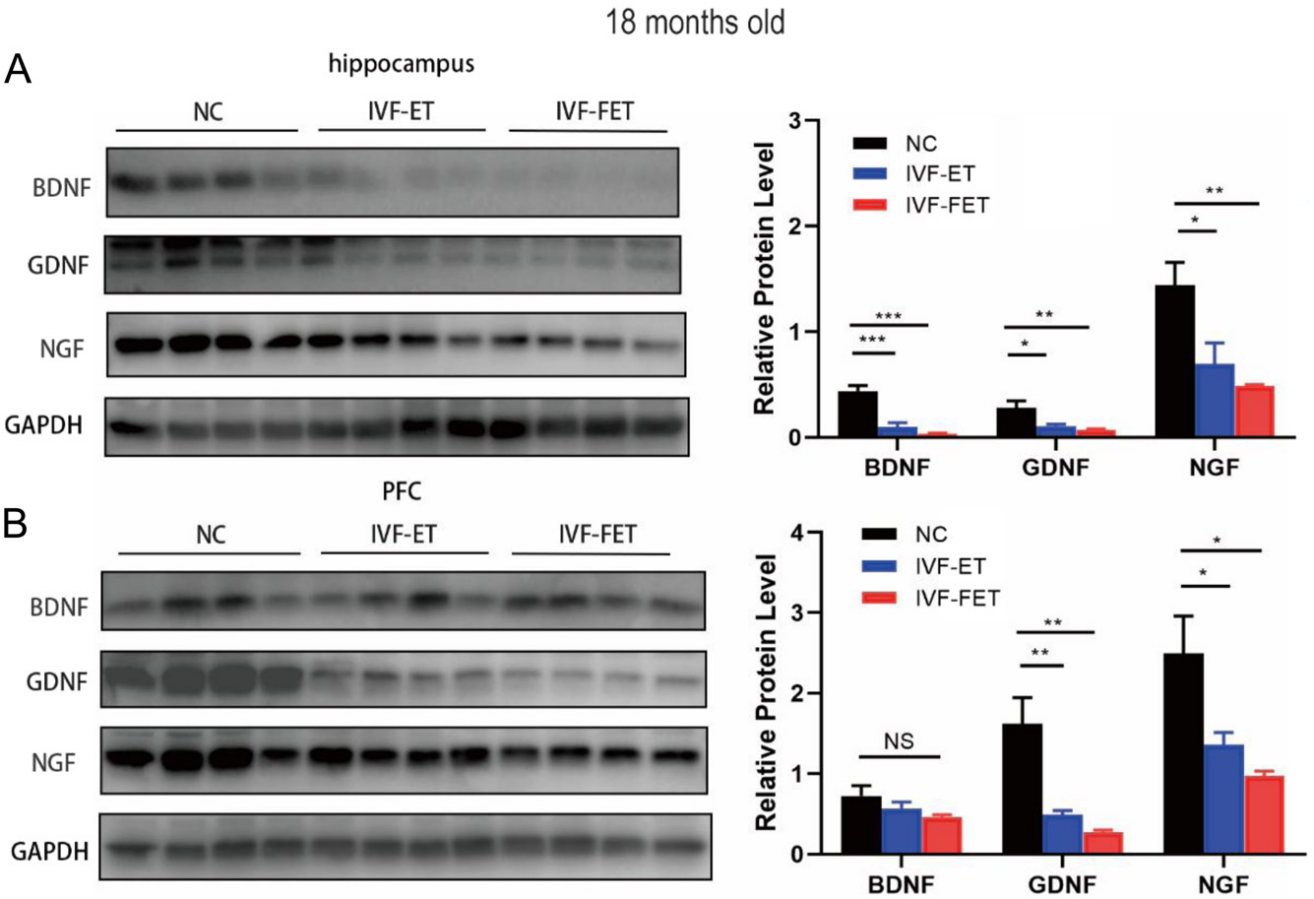
Western blotting results of three neurotrophins in the hippocampus and PFC of male mice offspring at 18 months old. Representative images of BDNF, GDNF, and NGF in the hippocampus (A) and PFC (B). All data are presented as the mean ± s.e.m. (*n* = 4 mice per group). NS: *P* > 0.05, *P < 0.05, **P < 0.01, ***P < 0.001.

We examined expression levels of three key neurotrophin proteins (BDNF, GDNF, and NGF) in the hippocampus and PFC of male mice offspring at 3 and 18 months old via Western blotting (Fig. 5 and Supplementary Fig. 3). The expression level of GDNF was significantly reduced in the hippocampus of male mice offspring in the IVF-FET group compared with NC and IVF-ET (Supplementary Fig. 3A), while no significant differences were found in the PFC in the three groups (Supplementary Fig. 3B). Meanwhile, we found that the protein expression levels of BDNF, GDNF, and NGF in the aged male mice offspring were decreased in the hippocampus from the offspring in the IVF-ET and IVF-FET groups (Fig. 5A), although there was no significant difference between the IVF-ET and IVF-FET groups. In the PFC, the expression levels of GDNF and NGF, but not BDNF, were reduced in the mice from both the IVF-ET and IVF-FET groups (Fig. 5B). The data suggested that decreased expression levels of BDNF, GDNF, and NGF were concordant with the increased anxiety and depression-like behaviours in aged male mice offspring conceived by ART.

### Transcriptional alterations in the hippocampus of aged offspring in the IVF-ET and IVF-FET groups

As there is a more severe reduction of neurotrophins in the hippocampus than in the PFC, we applied RNA-Seq to explore the gene expression profile changes in the hippocampus of aged offspring in IVF-ET and IVF-FET groups. And then, we selected 5 DEGs to validate the results of RNA-Seq. The results of qRT-PCR resonated with the data of RNA-Seq (Supplementary Fig. 4A).

Compared with NC group, 1625 genes were upregulated, and 159 genes were downregulated in IVF-ET group (supplementary Fig. 4B), meanwhile, 661 (480 genes upregulated and 181 genes downregulated) DEGs were identified in the IVF-FET group compared with the NC group (Supplementary Fig. 4C). Venn analysis showed that there are 305 DEGs (272 upregulated genes and 33 downregulated genes) between IVF-ET and IVF-FET groups compared with NC (Supplementary Fig. 4D and E).

**Figure 6:**
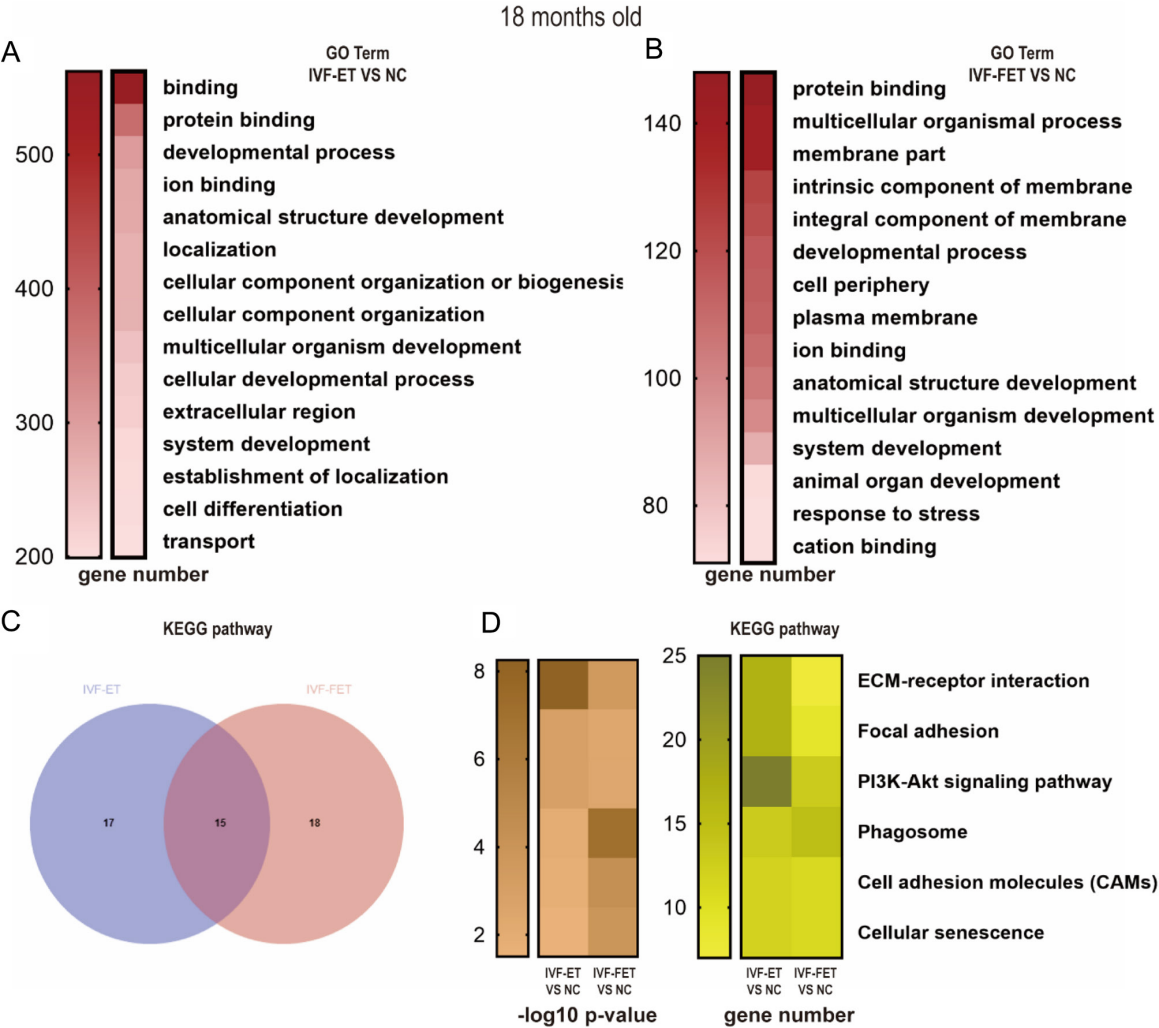
Gene ontology (GO) and Kyoto encyclopaedia of genes and genomes (KEGG) pathway enrichment analysis in the hippocampus of male mice offspring at 18 months old. The top 15 enriched GO terms ranked by the number of differential expression genes (DEGs) in the IVF-ET group (A) and IVF-FET group (B) compared with the NC group. (C) Venn diagram showed the enriched KEGG pathways terms in the two DEGs. (D) The heatmaps showed the *P* -values and number of DEGs in overlapped KEGG pathways terms (*n* = 3 mice per group).

Top 15 GO terms are shown in Fig. 6A (IVF-ET group vs NC group) and Fig. 6B (IVF-FET group vs NC group). Protein binding, developmental process, ion binding, anatomical structure development, multicellular organism development, and system development were listed in coexisted enriched top 15 GO terms analysis in the overlapped DEGs dataset between the IVF-ET and IVF-FET groups. Furthermore, DEGs were also analysed using KEGG database. We found that most enriched KEGG terms were overlapped in the two DEGs datasets (Fig. 6C). Among the 15 coexisted KEGG pathways, six pathways were associated with the nervous system, including ECM-receptor interaction, focal adhesion, PI3K/AKT signalling pathway, phagosome, cell adhesion molecules, and cellular senescence (Fig. 6D). Among the three groups, totally 73 genes were involved in the 6 KEGG pathways. Compared with the NC group, 55 and 33 genes were enriched in the IVF-ET and IVF-FET groups, respectively. All these genes were listed in Supplementary Tables 2 and 3.

## Discussion

Herein, we found that ART-conceived aged male mice offspring exhibited increased anxiety and depression-like behaviours compared with natural conception, extend the results from prior studies, which had implied increased risks of neurodevelopmental disease in the children born after ART (Strömberg *et al.* 2002, Zhu *et al.* 2009, Sandin *et al.* 2013).

Several mouse models have been established to investigate the effects of *in vitro* embryo culture (Ecker *et al.* 2004, Fernandez-Gonzalez *et al.* 2004), blastomere biopsy (Yu *et al.* 2009, Sampino *et al.* 2014, Wu *et al.* 2014), and intracellular sperm insemination (Fernandez-Gonzalez *et al.* 2008, Lewon *et al.* 2020), demonstrating that multiple procedures in ART enabled to increase anxiety level and deficit in learning abilities in offspring conceived by ART (Farquhar & Marjoribanks 2018). A previous study reported the reduced expression of MAO-A, CRFR2, and GABA markers in the hypothalamus and cortex, which were associated with high risk of anxiety and depression in mice conceived by IVF-ET compared with these in the natural pregnancy (Strata *et al.* 2015). Meanwhile, the levels of anxiety and depression were aggravating during ageing in mouse offspring conceived by IVF-ET (Fernandez-Gonzalez *et al.* 2004, Yu *et al.* 2011). These results are in line with our new findings. Meanwhile, our study first reported behavioural alterations in FET-conceived mice offspring. Mice offspring in IVF-FET group exhibited similar behaviroual alterations. Mice conceived by IVF-FET predisposed to depression, compared with IVF-ET. The potential harms of frozen and thawed process on the embryos may endow differential predisposition. Nevertheless, the exact molecular mechanisms still need further research in future.

BDNF, GDNF, and NGF are the most abundant neurotrophins that existed in CNS and involved in the pathophysiology of mood and other stress-related disorders (Bjorkholm & Monteggia 2016, Castren & Kojima 2017, von Bohlen Und Halbach & von Bohlen Und Halbach 2018). It has been demonstrated that the declination of these neurotrophins is highly correlated with the occurrence of anxiety and depression (Castren & Kojima 2017). At the age of 3 months old, ART-conceived male mice offspring did not exhibit alterations in behavioural assessment. Western blotting results revealed that GDNF was significantly decreased in the hippocampus of mice offspring in the IVF-FET groups compared with the other groups, while neurotrophins protein expression levels of PFC were similar in the three groups. At the age of 18 months old, ART-conceived male mice offspring exhibited increased anxiety and depression-like behaviours than natural conception. GDNF and NGF were significantly downregulated in the hippocampus and PFC, which might contribute to increase the anxiety and depression levels in the offspring from IVF-ET and IVF-FET groups. More severe alteration of neurotrophins protein expression levels of mice offspring at 18 months old than 3 months old was consistent with increased anxiety and depression-like behaviours in the aged offspring.

At the age of 3 months old, male mice offspring in IVF-FET group displayed decreased GDNF protein expression level in the hippocampus compared with NC and IVF-ET groups, although no behavioural alterations were observed. Reduced GDNF in CNS or peripheral serum could be a potential biomarker of mood disorder, especially for depression (Sun *et al.* 2019, Idemoto *et al.* 2021, Nedic Erjavec *et al.* 2021). Moreover, GDNF has been proven to promote the survival, differentiation, and neurite growth, most notably of dopaminergic neurons both *in vitro* and *in vivo* (Haniu *et al.* 1996, Nakajima *et al.* 2001, Consales *et al.* 2007). Thus, we speculate that the depression phenotype in aged ART-conceived mice offspring might be associated with the reduced GDNF and functions of dopaminergic neurons. At the age of 18 months old, ART-conceived male mice offspring displayed decreased BDNF protein expression level in the hippocampus compared with NC. Comparable expression level of BDNF in the PFC could be contributed to its complex and fruitful roles in the nervous system. For instance, BDNF is involved in hippocampus-dependent spatial learning and memory (Bjorkholm & Monteggia 2016, von Bohlen Und Halbach & von Bohlen Und Halbach 2018).

GO and KEGG analyses in DEGs provided the potential pathways associated with altered behaviours conceived by ART and advanced age. According to previous research, the overlapped six KEGG pathways in the overlapped DEGs datasets between the IVF-ET and IVF-FET groups were associated with the functions of nervous system. For instance, the ECM-receptor interaction pathway plays a pivotal role in the pathogenesis of several neurodegenerative and neuroinflammatory disorders, as the direct or indirect control of cellular processes such as adhesion, migration, differentiation, proliferation, and apoptosis (Kerrisk *et al.* 2014, Mardpour *et al.* 2019, Sun *et al.* 2020, Virtuoso *et al.* 2020, Su *et al.* 2021). Focal adhesion, phagosome, CAMs, and cellular senescence are enriched KEGG terms. These KEGG pathways interact with each other and participate in the ECM-receptor interaction pathway (Kerrisk *et al.* 2014, Mardpour *et al.* 2019, Sun *et al.* 2020, Virtuoso *et al.* 2020, Su *et al.* 2021). Furthermore, PI3K/AKT signalling pathway is strongly associated with anxiety and depression behaviours through hippocampal plasticity, cell growth, proliferation, survival, and metabolism (Gimenez-Llort *et al.* 2020, Neis *et al.* 2020, Palumbo *et al.* 2021).

Actually, we did not exactly interpret why the behavioural alterations occurred in the aged ART-conceived male mice offspring rather than adult offspring. Based on our analysis, we presume the ART procedure as ‘first hit’ in early life and ageing as ‘second hit’ for the offspring. The ‘second hit’ framework was a crucial theory to explain how lifelong health can be adversely affected by a series of ‘hits’ (Winett *et al.* 2016). In other words, when suffered with ageing, ART-conceived offspring seem biased towards anxiety and depression.

However, there are several limitations in our present study. First, ART procedures in mice are similar to humans, but does not recapture all procedures in the clinic. Different ovarian stimulation protocols and genetic backgrounds may affect the behavioural results in offspring for humans. Secondly, in addition to congenital background, there are many acquired aspects that might be related to anxiety and depression for humans, including home environment, educational background, life quality, income level, alcohol abuse, and stressful life events (Ransome *et al.* 2017, Czamara *et al.* 2021, Haugan *et al.* 2021, Sparling *et al.* 2021). Thirdly, we only assessed behaviours at 3 and 18 months old for offspring. We could not identify the onset time of anxiety and depression phenotypes in this study.

Nevertheless, our study had several strengths. The most important was that the mouse model excluded confounding effects of ovarian stimulation protocols and genetic backgrounds. Our current findings indicated that male offspring conceived by ART exhibited increased anxiety and depression-like behaviours when they were aged and lower expression of three neurotrophic factors which might contribute to the phenotypes occurred. This work paves the way to further investigate the precise mechanisms underpinning anxiety and depression in aged male offspring conceived by ART.

## Supplementary Material

Supplementary Fig. 1 Behavioral results of male mice offspring at 3 months old. (A-C) Total distance travelled (A), average velocity (B) and percentage of activity (C) in the OFT. (D-F) Distance (D), time (E) and entries (F) in the centre zone in the OFT. (G-I) Distance (G), entries (H) and time (I) in open arms in the EPM. (J) Immobility time in the TST. (K) Latency time to the platform on training days in the MWM. (L) Distance spent in the target quadrant on the test day in the MWM. All data are presented as the mean ± SEM (n= 7 mice per group). NS: P＞0.05.

Supplementary Fig. 2 MWM results of male mice offspring at 18 months old. (A) Latency time to the platform on training days in the MWM. (B) Distance in the target quadrant on the test day in the MWM. All data are presented as the mean ± SEM (n= 7 mice per group). NS: P>0.05.

Supplementary Fig.3 Western blotting results of three neurotrophins in the hippocampus and PFC of male mice offspring at 3 months old. Representative images of BDNF, GDNF and NGF in the hippocampus(A) and PFC (B). All data are presented as the mean ± SEM (n=4 mice per group). NS: P>0.05, **: P<0.01.

Supplementary Fig.4 Transcriptome profile for hippocampus of male mice offspring at 18 months old in the IVF-ET and IVF-FET groups. (A) The qRT-PCR validation of DEGs in RNA-seq. The relative mRNA expression levels in the hippocampus of offspring were expressed as the mean ± SEM (n= 6 mice per group). (B, C) Scatterplot showing the number of genes upregulated (red) and downregulated (green) in hippocampus of offspring in the IVF-ET (B) and IVF-FET (C) groups compared with the NC group (n=3 mice per group). (D, E) Venn diagrams showing upregulated genes (D) and downregulated genes (E) in hippocampus of offspring in the IVF-ET and IVF-FET groups compared with the NC group (n=3 mice per group).

Supplementary Table 1 Primers are used for RT-qPCR. Sequences are printed in the 5’ to 3’direction.

Supplementary Table 2 KEGG pathway enrichment analysis of DEGs in IVF-ET and IVF-FET groups compared with NC.

Supplementary Table 3 DEGs in selected KEGG enrichment pathways.

## Declaration of interest

The authors declare that there is no conflict of interest that could be perceived as prejudicing the impartiality of the research reported.

## Funding

This work was supported by the National Key Research and Development Project (no. 2017YFC1001300 to H-F H, no. 2018YFC1004900 to W-L Z), the National Natural Science Foundation of China http://dx.doi.org/10.13039/501100001809 (no. 81661128010 to H-F H, no. 81701504 to W-L Z, no. 82088102 to H-F H), the Shanghai Municipal Science and Technology Innovation Action Plan (no. 20XD1424100 to Y-T W), Shanghai Shenkang Hospital Developmental Center (no. SHDC12018X17 to Y-T W), General Program of Shanghai Municipal Commission of Health and Family Planning (no. 201840210), General Program of Shanghai Jiaotong University Medical Engineering Cross Fund (no. YG2019GD04 to Y-T W), Chinese Academy of Medical Sciences http://dx.doi.org/10.13039/501100000691 Research Unit (no. 2019RU056), Shanghai Jiao Tong University http://dx.doi.org/10.13039/501100004921, CAMS Innovation Fund for Medical Sciences (CIFMS) (no. 2019-I2M-5-064) and Shanghai Municipal Key Clinical Specialty, Shanghai, China.

## Author contribution statement

N-X Q, Y-R Z, W-H S, and Z-Y Z designed and performed experiments and analysed data. N-X Q, W-L Z, and Z-Y Z wrote and edited the manuscript. K-X Z and W-H S contributed to data analysis. C-J Y, X L, Z-H D, Y-T M, J-L Y, and C-L Z contributed to conducting the experiment. X-M L, J-Z S, and G-L D contributed to the study design, discussion, and conducted experiments. Y-T W and H-F H designed and supervised the research, contributed to discussion, and edited the manuscript. H-F H is the guarantor of this work and has full access to all data in the study and takes responsibility for the integrity and accuracy of data analyses.
