# Supplementary figures and images for: Anxiety and depression-like behaviours are more frequent in aged male mice conceived by ART compared with natural conception
Source: Reproduction. 2021 Oct 4;162(6):437–48. doi: 10.1530/REP-21-0175 (PMC8630775; doi:10.1530/REP-21-0175)

Supp Figure 1

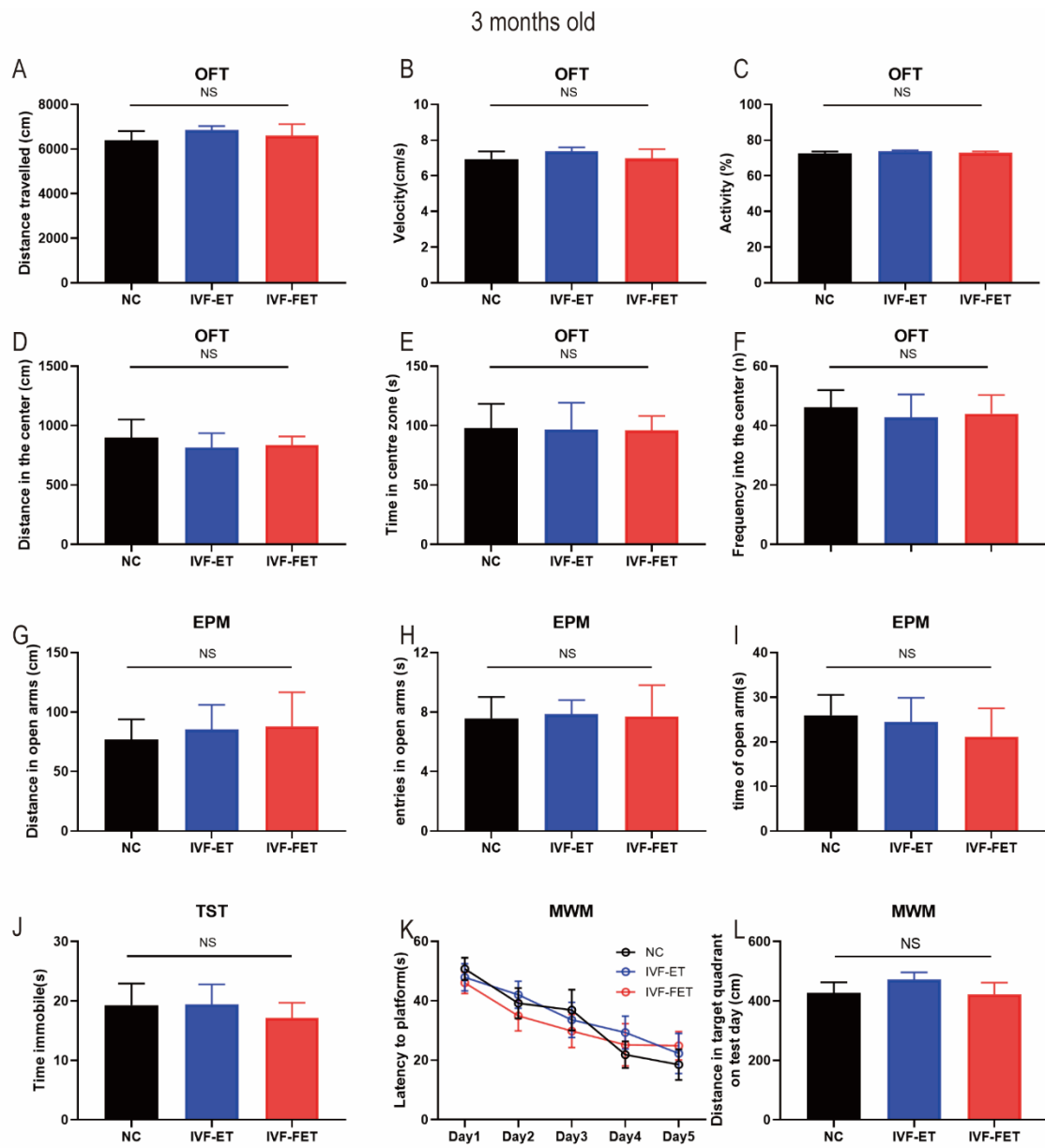

Supplement: Supplementary Fig. 1 Behavioral results of male mice offspring at 3 months old. (A-C) Total distance travelled (A), average velocity (B) and percentage of activity (C) in the OFT. (D-F) Distance (D), time (E) and entries (F) in the centre zone in the OFT. (G-I) Distance (G), entries (H) and time (I) [file supplementary_figure_1.pdf]

Supp figure 2

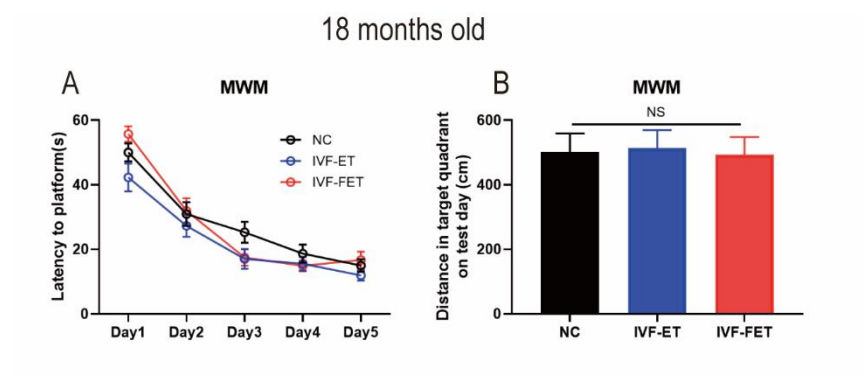

Supplement: Supplementary Fig. 2 MWM results of male mice offspring at 18 months old. (A) Latency time to the platform on training days in the MWM. (B) Distance in the target quadrant on the test day in the MWM. All data are presented as the mean ± SEM (n= 7 mice per group). NS: P>0.05. [file supplementary_figure_2.pdf]

Supp figure 3

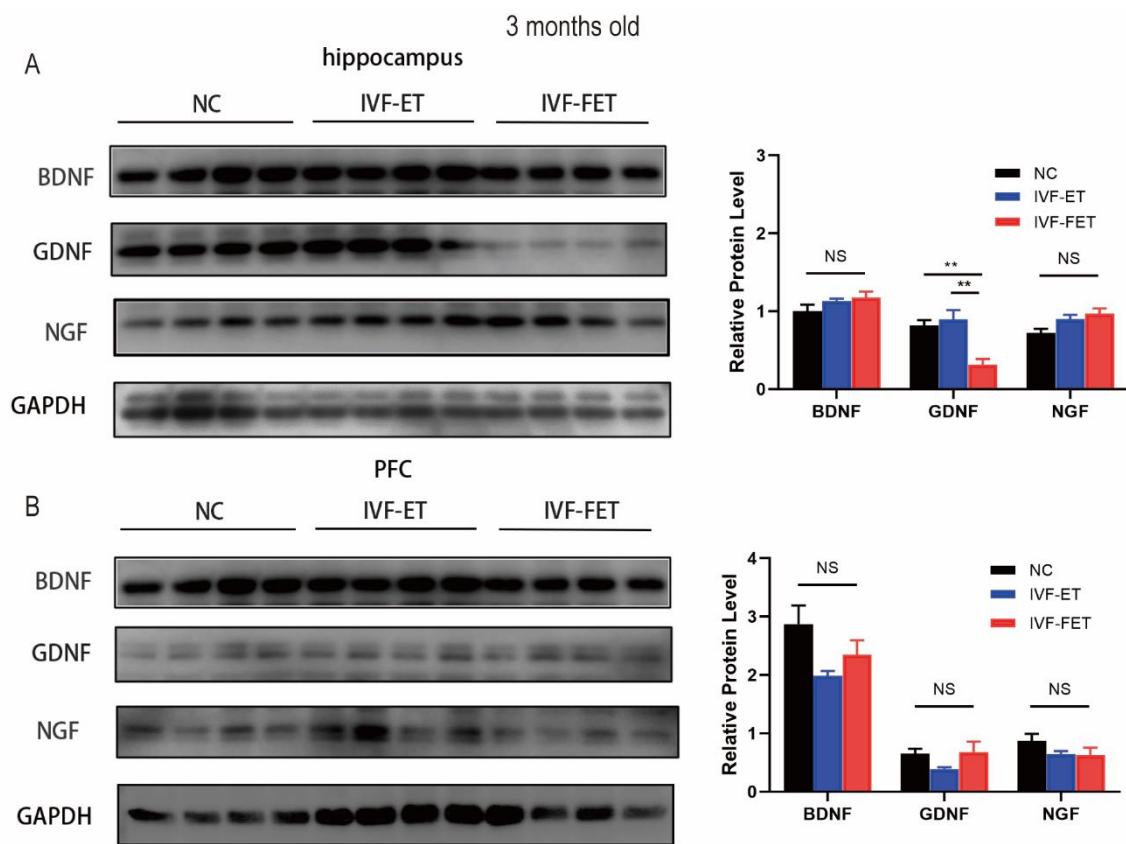

Supplement: Supplementary Fig.3 Western blotting results of three neurotrophins in the hippocampus and PFC of male mice offspring at 3 months old. Representative images of BDNF, GDNF and NGF in the hippocampus(A) and PFC (B). All data are presented as the mean ± SEM (n=4 mice per group). NS: P>0.05, **: P<0.01. [file supplementary_figure_3.pdf]

Supp figure 4

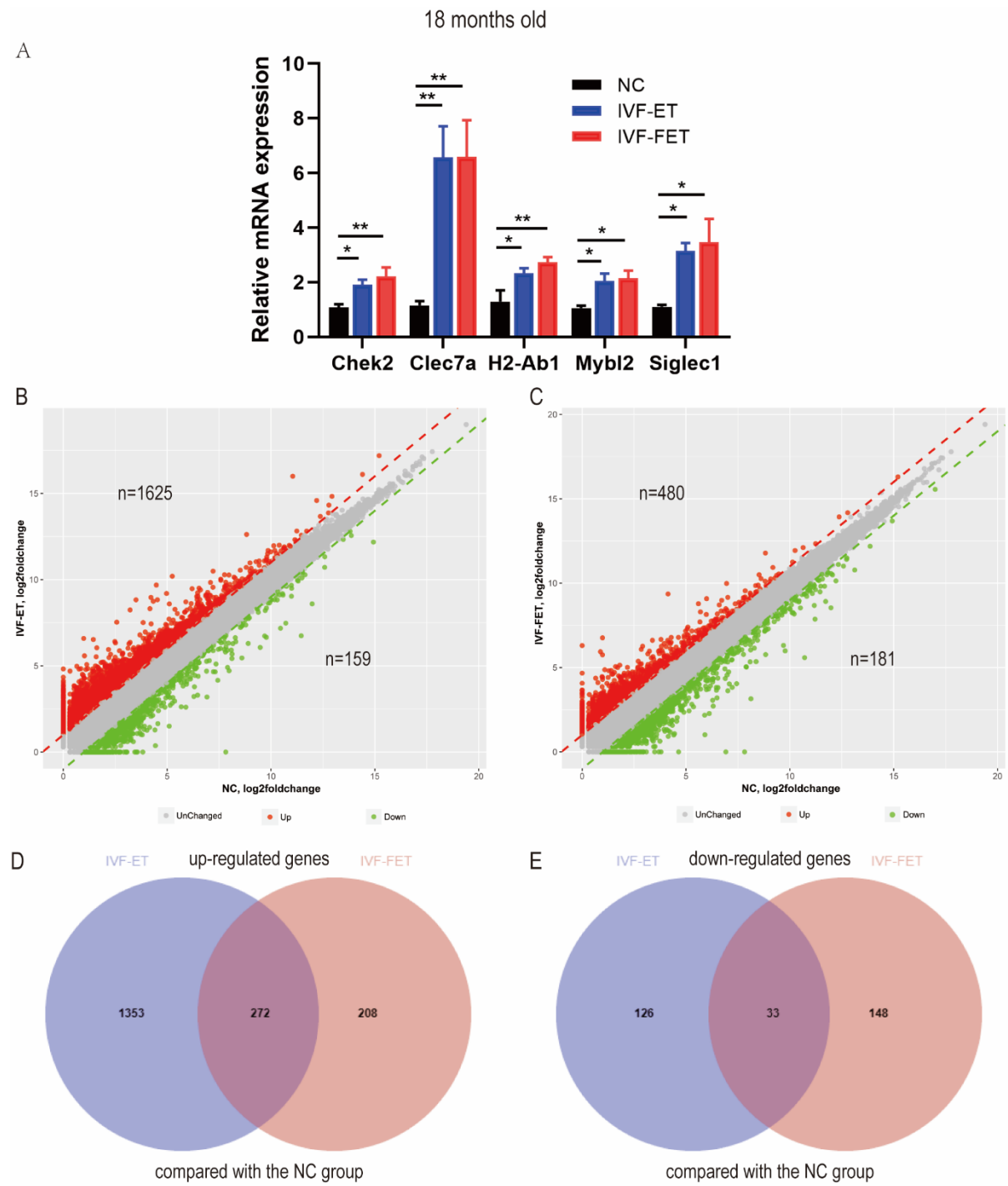

Supplement: Supplementary Fig.4 Transcriptome profile for hippocampus of male mice offspring at 18 months old in the IVF-ET and IVF-FET groups. (A) The qRT-PCR validation of DEGs in RNA-seq. The relative mRNA expression levels in the hippocampus of offspring were expressed as the mean ± SEM (n= 6 mice per group [file supplementary_figure_4.pdf]
