# Supplementary material for: Anxiety and depression-like behaviours are more frequent in aged male mice conceived by ART compared with natural conception
Source: Reproduction. 2021 Oct 4;162(6):437–48. doi: 10.1530/REP-21-0175 (PMC8630775; doi:10.1530/REP-21-0175)
Supplement: Supplementary Table 1 Primers are used for RT-qPCR. Sequences are printed in the 5’ to 3’direction. [file supplementary_table_1.pdf]

Supplementary Table 1

Primers are used for RT-qPCR. Sequences are printed in the 5’ to 3’direction.

Transcript	Primers
GAPDH	F: AGGTCGGTGTGAACGGATTTG<br>R: GGGGTCGTTGATGGCAACA
Chek2	F: GATCATTAGCAAGCGGAGGTT<br>R: CACCACCCGGTCAAATAGTTC
Clec7a	F: GACTTCAGCACTCAAGACATCC<br>R: TTGTGTCGCCAAAATGCTAGG
H2-Ab1	F: ACAGCTTATTAGGAATGGGGACT<br>R: CACGGTGATGGGACTCTTCA
Mybl2	F: AGGACGTGGACTCAGACCTC<br>R: CTGTGCGGTTAGGAAAGTGAC
Siglec1	F: CAGGGCATCCTCGACTGTC<br>R: GGAGCATCGTGAAGTTGGTTG
