# Supplementary material for: Anxiety and depression-like behaviours are more frequent in aged male mice conceived by ART compared with natural conception
Source: Reproduction. 2021 Oct 4;162(6):437–48. doi: 10.1530/REP-21-0175 (PMC8630775; doi:10.1530/REP-21-0175)
Supplement: Supplementary Table 2 KEGG pathway enrichment analysis of DEGs in IVF-ET and IVF-FET groups compared with NC. [file supplementary_table_2.pdf]

Supplementary Table 2 KEGG pathway enrichment analysis of DEGs in IVF-ET and IVF-FET groups compared with NC.

| Pathway name | Pathway class | IVF-ET VS NC | | IVF-FET VS NC | |
|---|---|---|---|---|---|
| | | Gene count | gene | Gene count | gene |
| ECM-receptor interaction | Signaling molecules and interaction | 17 | Col9a1, Sdc1, Col1a1, Col9a3, Col4a5, Itga10, Col4a3, Lama5, Col1a2, Col6a1, Itgb7, Cd36, Col6a2, Col4a6, Col4a4, Col6a4, Thbs3 | 7 | Col6a6, Itga11, Comp, Lama1, Itga10, Col6a4, Itgb7 |
| Focal adhesion | Cellular community - eukaryotes | 17 | Col9a1, Col1a1, Col9a3, Col4a5, Itga10, Col4a3, Lama5, Col1a2, Flna, Col6a1, Itgb7, Col6a2, Cav3, Col4a6, Col4a4, Col6a4, Thbs3 | 9 | Col6a6, Itga11, Comp, Met, Lama1, Itga10, Col6a4, Myl9, Itgb7 |
| PI3K-Akt signaling pathway | Signal transduction | 25 | Col9a1, Ntrk1, Igf2, Tlr2, Fgf16, Col1a1, Ins2, Fgf17, Col9a3, Col4a5, Itga10, Col4a3, Creb3l4, Il3ra, Lama5, Col1a2, Col6a1, Itgb7, Prlr, Col6a2, Fgf5, Col4a6, Col4a4, Col6a4, Thbs3 | 13 | Col6a6, Itga11, Nras, Comp, Met, Lama1, Igf2, Itga10, Col6a4, Il3ra, Fgf15, Itgb7, Prlr |
| Phagosome | Transport and catabolism | 13 | Clec7a, Tlr2, H2-M10.2, Atp6v1c2, Pla2r1, H2-Q1, Rilp, Cd36, Atp6v0c, Cybb, Thbs3, H2-M5, H2-Ab1 | 15 | Gm11127, H2-Ab1, C3, Comp, H2-Q4, H2-M5, Atp6v1b1, Pla2r1, H2-T10, Clec7a, H2-Q7, Cybb, H2-Q1, H2-Q6, H2-Aa |
| Cell adhesion molecules (CAMs) | Signaling molecules and interaction | 12 | Sdc1, H2-M10.2, Cldn2, H2-Q1, Cldn3, Itgb7, Cdh1, Cldn9, Siglec1, Cdh3, H2-M5, H2-Ab1 | 11 | Gm11127, H2-Ab1, H2-Q4, H2-M5, H2-T10, H2-Q7, H2-Q1, Siglec1, Itgb7, H2-Q6, H2-Aa |
| Cellular senescence | Cell growth and death | 12 | H2-M10.2, Calml4, Mybl2, H2-Q1, Trpv4, Chek1, Chek2, Rbl1, Slc25a31, Serpine1, Traf3ip2, H2-M5 | 11 | Gm11127, Nras, H2-Q4, H2-M5, H2-T10, H2-Q7, Nfatc4, H2-Q1, H2-Q6, Chek2, Mybl2 |
