# Supplementary material for: Anxiety and depression-like behaviours are more frequent in aged male mice conceived by ART compared with natural conception
Source: Reproduction. 2021 Oct 4;162(6):437–48. doi: 10.1530/REP-21-0175 (PMC8630775; doi:10.1530/REP-21-0175)
Supplement: Supplementary Table 3 DEGs in selected KEGG enrichment pathways. [file supplementary_table_3.pdf]

Supplementary Table 3 DEGs in selected KEGG enrichment pathways.

Gene symbol	Gene name	IVF-ET VS NC		IVF-FET VS NC	
		Log2 FC	P value	Log2 FC	P value
Chek2	checkpoint kinase 2	1.15	0.0019	1.17	0.0026
Clec7a	C-type lectin domain family 7, member a	1.53	0.0154	1.55	0.0228
Col6a4	collagen, type VI, alpha 4	1.09	0.0038	1.85	0.0002
Cybb	cytochrome b-245, beta polypeptide	1.56	0.0082	1.49	0.0165
H2-Ab1	histocompatibility 2, class II antigen A, beta 1	1.20	0.0075	1.36	0.0054
H2-M5	histocompatibility 2, M region locus 5	1.73	< 0.0001	1.11	0.0166
H2-Q1	histocompatibility 2, Q region locus 1	1.59	0.0064	1.77	0.0053
Igf2	insulin-like growth factor 2	1.84	0.0142	1.12	0.0371
Il3ra	interleukin 3 receptor, alpha chain	1.05	0.0361	1.32	0.0090
Itga10	integrin, alpha 10	1.69	< 0.0001	1.28	0.0021
Itgb7	integrin beta 7	1.08	0.0212	1.04	0.0356
Mybl2	myeloblastosis oncogene-like 2	1.30	0.0009	1.09	0.0189
Pla2r1	phospholipase A2 receptor 1	1.82	0.0007	1.42	0.0235
Prlr	prolactin receptor	2.97	0.0016	-2.16	< 0.0001
Siglec1	sialic acid binding Ig-like lectin 1, sialoadhesin	2.88	0.0021	3.33	0.0016
Atp6v0c	ATPase, H+ transporting, lysosomal V0 subunit C	-1.01	< 0.0001	/	NS
Atp6v1c2	ATPase, H+ transporting, lysosomal V1 subunit C2	1.05	0.0122	/	NS
Calml4	calmodulin-like 4	2.29	< 0.0001	/	NS
Cav3	caveolin 3	-1.57	0.0222	/	NS
Cd36	CD36 antigen	2.01	0.0012	/	NS
Cdh1	cadherin 1	2.53	< 0.0001	/	NS
Cdh3	cadherin 3	4.43	0.0080	/	NS
Chek1	checkpoint kinase 1	1.09	0.0109	/	NS
Cldn2	claudin 2	4.41	0.0001	/	NS
Cldn3	claudin 3	1.98	0.0025	/	NS
Cldn9	claudin 9	3.28	0.0005	/	NS

Col1a1	collagen, type I, alpha 1	1.14	0.0001	/	NS
Col1a2	collagen, type I, alpha 2	1.20	0.0136	/	NS
Col4a3	collagen, type IV, alpha 3	2.70	0.0033	/	NS
Col4a4	collagen, type IV, alpha 4	2.27	0.0006	/	NS
Col4a5	collagen, type IV, alpha 5	1.17	< 0.0001	/	NS
Col4a6	collagen, type IV, alpha 6	1.04	0.0029	/	NS
Col6a1	collagen, type VI, alpha 1	1.83	0.0085	/	NS
Col6a2	collagen, type VI, alpha 2	1.31	0.0150	/	NS
Col9a1	collagen, type IX, alpha 1	1.46	0.0039	/	NS
Col9a3	collagen, type IX, alpha 3	1.81	0.0066	/	NS
Creb3l4	cAMP responsive element binding protein 3-like 4	1.31	0.0371	/	NS
Fgf16	fibroblast growth factor 16	-1.40	0.0090	/	NS
Fgf17	fibroblast growth factor 17	2.59	0.0094	/	NS
Fgf5	fibroblast growth factor 5	-1.01	0.0005	/	NS
Flna	filamin, alpha	1.12	0.0002	/	NS
H2-M10.2	histocompatibility 2, M region locus 10.2	1.69	0.0163	/	NS
H2-M5	histocompatibility 2, M region locus 5	1.73	< 0.0001	/	NS
Ins2	insulin II	2.96	0.0057	/	NS
Lama5	laminin, alpha 5	1.08	0.0215	/	NS
Ntrk1	neurotrophic tyrosine kinase, receptor, type 1	3.33	0.0007	/	NS
Rbl1	retinoblastoma-like 1 (p107)	1.08	0.0075	/	NS
Rilp	Rab interacting lysosomal protein	1.10	0.0051	/	NS
Sdc1	syndecan 1	1.09	0.0244	/	NS
Serpine1	serine (or cysteine) peptidase inhibitor, clade E, member 1	1.44	0.0046	/	NS
Slc25a31	solute carrier family 25 (mitochondrial carrier; adenine nucleotide translocator), member 31	2.39	0.0467	/	NS
Thbs3	thrombospondin 3	1.22	< 0.0001	/	NS
Tlr2	toll-like receptor 2	5.67	1.3396	/	NS
Traf3ip2	TRAF3 interacting protein 2	8.23	1.2360	/	NS
Trpv4	transient receptor potential cation channel, subfamily V, member 4	2.45	0.0007	/	NS
Atp6v1b1	ATPase, H+ transporting,	/	NS	2.21	0.0322

	lysosomal V1 subunit B1				
C3	complement component 3	/	NS	-1.53	0.0179
Col6a6	collagen, type VI, alpha 6	/	NS	-1.48	0.0140
Comp	cartilage oligomeric matrix protein	/	NS	1.18	0.0272
Fgf15	fibroblast growth factor 15	/	NS	-2.49	0.0301
Gm11127	predicted gene 11127	/	NS	2.33	0.0389
H2-Aa	histocompatibility 2, class II antigen A, alpha	/	NS	1.34	0.0018
H2-Q4	histocompatibility 2, Q region locus 4	/	NS	1.11	< 0.0001
H2-Q6	histocompatibility 2, Q region locus 6	/	NS	1.44	0.0001
H2-Q7	histocompatibility 2, Q region locus 7	/	NS	1.30	0.0012
H2-T10	histocompatibility 2, T region locus 10	/	NS	1.19	0.0001
Itga11	integrin alpha 11	/	NS	1.00	0.0012
Itgb7	integrin beta 7	/	NS	1.04	0.0356
Lama1	laminin, alpha 1	/	NS	1.01	0.0013
Met	met proto-oncogene	/	NS	-1.50	0.0039
Myl9	myosin, light polypeptide 9, regulatory	/	NS	1.01	0.0010
Nfatc4	nuclear factor of activated T cells, cytoplasmic, calcineurin dependent 4	/	NS	1.05	0.0214
Nras	neuroblastoma ras oncogene	/	NS	1.11	0.0232

Log2 FC: log2 fold change, NS: P > 0.05.
